# Epigenetic modulations of immune cells: from normal development to tumor progression

**DOI:** 10.7150/ijbs.88327

**Published:** 2023-10-02

**Authors:** Yuanchun Xu, Zongsheng He, Jing Du, Ziqiang Chen, John W. M. Creemers, Bin Wang, Fan Li, Yaling Wang

**Affiliations:** 1 Department of General Surgery, Daping Hospital, Army Medical University, Chongqing, China.; 2 Department of Gastroenterology, Daping Hospital, Army Medical University, Chongqing, China.; 3 Department of Human Genetics, KU Leuven, Leuven, Belgium.; 4 Department of nursing, Daping Hospital, Army Medical University, Chongqing, China.

**Keywords:** Innate immune cells, adaptive immune cells, DNA methylation, histone modifications, chromatin remodeling, RNA-associated modulations, cancer treatment

## Abstract

The dysfunction of immune cell development often impairs immunological homeostasis, thus causing various human diseases. Accumulating evidence shows that the development of different immune cells from hematopoietic stem cells are highly fine-tuned by different epigenetic mechanisms including DNA methylation, histone modifications, chromatin remodeling and RNA-related regulations. Understanding how epigenetic regulators modulate normal development of immune cells contributes to the identification of new strategies for various diseases. Here, we review recent advances suggesting that epigenetic modulations can orchestrate immune cell development and functions through their impact on critical gene expression. We also discuss the aberrations of epigenetic modulations in immune cells that influence tumor progression, and the fact that underlying mechanisms affect how epigenetic drugs interfere with tumor progression in the clinic.

## Introduction

Epigenetic modifications refer to heritable alterations that take place in gene expression without altering the DNA nucleotide sequence. The underlying mechanisms of epigenetic modifications mainly include DNA methylation, histone modifications, chromatin remodeling and RNA-related mechanisms 1. Such epigenetic mechanisms could result in a wide variety of cellular functions, morphogenesis and physiological processes by regulating related gene expression. Not surprisingly, epigenetic modifications are closely associated with human pathophysiology. For instance, epigenetic dysregulation directly influences the development of autoimmunity by regulating immune cell functions 2.

The immune system can be classified into two types: innate immunity and adaptive immunity, which are pivotal for the human body to respond to numerous signals in the microenvironment. The appropriate differentiation and activation of immune cells into diverse functional states is crucial for immunological homeostasis. Recently, genome-wide epigenetic landscapes have been established for several immune cells including tissue-resident macrophages 3 and T cells 4, which provide evidence that epigenetic modifications have been linked to multiple aspects of immune cell development, activation, and differentiation by impacting expression of critical genes.

Immune dysfunction due to abnormal epigenetic modifications has been closely associated with tumor progression. For example, SETDB1-dependent H3K9me3 represses the expression of T helper 1(Th1) cell-associated genes in CD4^+^ T cells by repressing a repertoire of ERVs that have been exapted into *cis*-regulatory modules to shape and control the Th1 gene network 5, which contributes to the generation of immune-permissive status in the tumor microenvironment. In addition, epigenetic dysfunction of tumor cells could interfere with the functioning of the immune system, which in turn promotes tumor initiation, progression and metastasis. Therefore, epigenetic reprogramming has been recognized as one of the hallmarks of cancer 6. Targeting the epigenetic alterations in cancer cells or the tumor microenvironment (TME) with epigenetic-associated drugs could switch an immune-suppressive to an immune-permissive state, allowing tumor cells to be attacked by immune cells. Knowing how epigenetic alterations affect immune cell development and function could contribute to identifying novel targets for cancer treatment.

## Epigenetic classification

**Figure 1:**
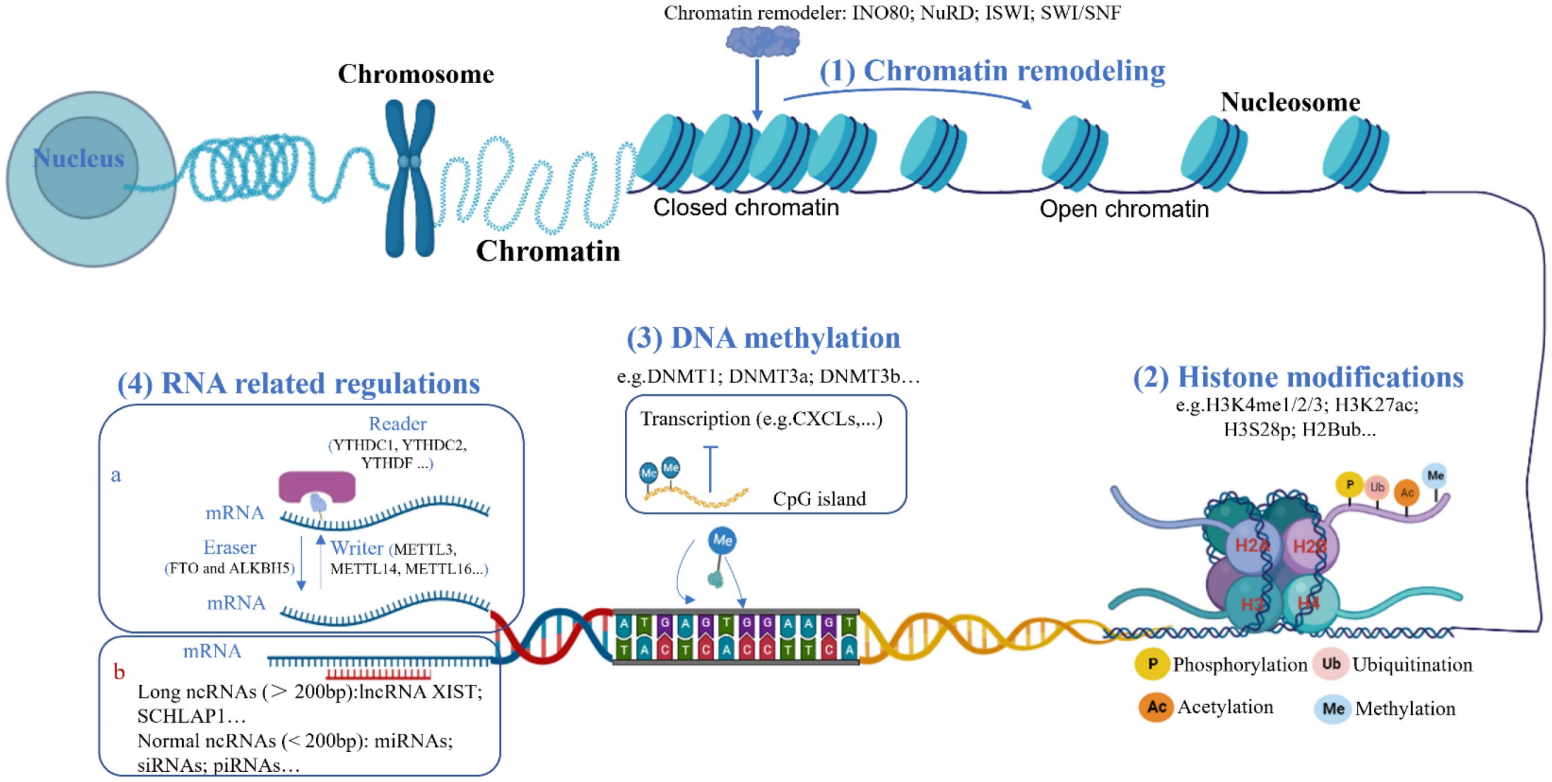
** Schematic model of four epigenetic modifications.** Chromatin remodeling allows TFs or other DNA binding proteins to access DNA and control gene expression by the rearranging chromatin from a closed state to an open state. There are four major types of Chromatin remodeler: INO80, NuRD, ISWI and SWI/SNF family. The effects of histone modifications on gene expression could differ from type to type. We list four common types of histone modifications including histone acetylation (Ac), histone methylation (Me), histone phosphorylation (P) and histone ubiquitination (Ub). DNA methylation often exists in GC-rich areas of the human genome called CpG islands, which can be methylated by DNMTs, resulting in repression of transcription of genes. In contrast, DNA demethylation function as opposite effects on the transcription of genes. When DNA transcribed into RNAs, the RNAs can be methylated in a reversible manner, which regulates their translation, stability and decay. Furthermore, many DNA sequences are not transcribed into mRNAs but are transcribed as ncRNAs. Based on the length, ncRNAs can be divided into short and long ncRNAs (lncRNAs). Short ncRNAs are less than 200 bp in length, including microRNAs (miRNAs), small interfering RNAs (siRNAs), PIWI-interacting RNAs (piRNAs), etc. lncRNAs are more than 200 bp in length. They target the 3'-UTR of mRNA, thus causing gene silencing.

The term “epigenetics” was first described by Conrad H Waddington in 1942. The word is derived from the Greek word “epigenesis”, and initially described the effects of genetic processes on development. Now, the term epigenetics is used to describe the process by which changes mediating heritable patterns of gene expression that occur without a change in DNA sequence. The mechanisms responsible for these changes can be broadly classified into four major categories: DNA methylation, histone modifications, chromatin conformation and RNA related mechanisms (Figure 1).

### Chromatin remodeling

Chromatin remodeling is a complex process regulating nucleosomes to gain access to the target chromatin, which are performed by different chromatin remodelers in an ATP-dependent manner. Based on the structure, chromatin remodeler can be classified into four families in eukaryotes: the inositol 80 (INO80), the Mi-2/nucleosome remodeling and histone deacetylation (NuRD) family, the imitation-switch (ISWI) family, and the switching defective/sucrose non-fermenting (SWI/SNF) family 7. All these families contain one or two distinct SWI2/SNF2-type catalytic ATPases that provide the energy for disrupting contacts between the DNA and histones by hydrolyzing ATP, leading to nucleosome disassembly. The re-organized nucleosome plays an important role in regulating transcriptional processes including transcription initiation, elongation and termination, by either causing partial disassembly of histone octamers in regulatory regions or moving the intact histone octamer to a new region on the genomic DNA. In addition, chromatin remodelers are associated with other cellular functions like DNA repair and chromatin maintenance.

### Epigenetic modulations associated with histone proteins

Histone is the basic component of the nucleosome that is composed of an octamer containing the four core histone proteins (H3, H4, H2A, H2B), which is further wrapped by a 147-base pairs segment of DNA. As with DNA methylation, histone modifications can regulate its availability to the transcriptional machinery without altering the DNA sequence. Normally, histone modifications can be achieved by different enzymes at the histone N-terminal tails containing certain amino acids such as lysine, arginine, serine, threonine, tyrosine, etc. Different histone modifications could have distinct effects on gene expression. For instance, H3K27ac modification induces gene expression, while H3K27me modification represses gene expression. So far numerous histone modifications have been identified, which show distinct effects on gene expression (Table 1). In addition, histone modifications play an important role in regulating interaction of different histones or histone-DNA. Moreover, histone modifications might provide or remove binding sites for certain protein complexes, like histone-modifying enzymes and chromatin remodeling complexes 8. As a result, histone modifications modulate gene expression by influencing the chromatin structure and protein-protein interactions. Here, we briefly introduce the four most common histone post-translational modification activities.

#### Histone acetylation

The histone acetylation state is regulated by two kinds of enzymes with opposing functions: histone acetyltransferases (HATs) and histone deacetylases (HDACs). So far three major groups of HATs have been identified: GNAT (HAT1, GCN5, PCAF), MYST (Tip60, MOF, MOZ, MORF, HBO1), and p300/CBP 9. HATs add an acetyl group from acetyl-CoA to the side chain of the lysine residues on histone tails, neutralizing the positively charged lysine, which breaks the interaction between histones and the negatively charged nucleosomal DNA 10. This in turn facilitates the opening of chromatin to activate transcription. In addition, acetylated lysines of chromatin contribute to the opening of chromatin by attracting various bromodomain-containing transcription factors (TFs). Furthermore, HATs can regulate protein stability, DNA binding and protein-protein interaction by catalyzing the acetylation of a wide range of non-histone proteins like oncoprotein p53 and Myc^11^.

In contrast, deacetylation of histones by the HDACs can reduce the accessibility of TFs by forming a closed chromatin conformation. HDACs are composed of four major classes (class I, II, III and IV). Class I, II, and IV are Zn^2+^-dependent, while class III/sirtuins are nicotinamide adenine dinucleotide (NAD)-dependent 12. Class I (HDACs 1, 2, 3, and 8) is characterized by a ubiquitous nuclear expression in all tissues; Class II (HDACs 4, 5, 6, 7, 9 and 10) exhibits tissue-specific expression and can transport between the nucleus and cytoplasm; different class III/sirtuins (SIRT1-7) members might localize in the cytoplasm, nucleus or mitochondria; class IV only contains HDAC11, and localizes predominantly to the cell nucleus 12. Similar to HATs, HDACs can also catalyze different non-histone substrates like p53 and β-catenin. Dysregulations of HDACs activity have recently been discovered in many human diseases.

#### Histone methylation

Histone methylation is a process of the addition of methyl groups to lysine (K) and arginine (R) residues on histone tails including histone H3 and H4. This process is tightly modulated by lysine methyltransferases (KMTs) and protein arginine methyltransferases (PaRMTs). KMTs are comprised of six major classes (KMT1-6). The KMT1 family includes at least four members including GLP, SUV39H1/2, G9a and SETDB1, which typically methylate H3K9^13^. The KMT2 family includes three subgroups, which functions normally within the macromolecular complex known as complex of proteins associated with SET1 (COMPASS) to methylate H3K4. The KMT3 family comprises NSD1, NSD2, and NSD3 and primarily deposits methyl group on H3K36. The KMT4 family has one sole member DOT1L, adding methyl group on H3K79^14^. The KMT5 family significantly methylates H4K20 through two enzymes: the PR-SET7 and SUV4-20H1/2^15^. The KMT6 family methylates histone H3K27 by two functionally redundant members EZH1 and EZH2, two functionally redundant enzymes, for H3K27 methylation 16. So far, nine PRMTs have been discovered in mammals. On the basis of the type of methyl arginine they produce, PRMTs are classified into three subgroups: Type I PRMTs (PRMT1, 2, 3, 4, 6, and 8) generate ω-N^G^-monomethyl arginine (MMA) and ω-N^G^,N^G^-asymmetric dimethyl arginine (ADMA), Type II PRMTs (PRMT5 and 9) produce MMA and ω-N^G^,N'^G^-symmetric dimethyl arginine (SDMA), and Type III PRMT (PRMT7) produces only MMA^17^.

#### Histone phosphorylation

Histone phosphorylation is a process, in which phosphate groups are added to the serine, threonine and tyrosine residues of nucleosomal histone tails by kinases. Conversely, the phosphorylated histones can be dephosphorylated by phosphatases. Phosphorylated histones have been reported to be involved in different cellular functions like DNA damage repair, chromatin compaction and transcription regulation. For instance, Lau et al. reported that phosphorylation of H2B serine 32 has been connected to the expression of proto-oncogenes like c-Jun and c-Fos 18. Furthermore, histone phosphorylation can work together with other histone modifications, generating the platform for mutual interactions among them. For instance, H3 serine 28 phosphorylation at gene promoters is thought to induce demethylation and acetylation of the adjacent K27 residue at these loci, thereby promoting transcription 19.

#### Histone ubiquitination

Ubiquitination refers to the process of addition of the ubiquitin (Ub), a 76-amino acid protein, to the substrate or itself to form different poly Ub chains via its C-terminus. This process contains a three-step enzymatic cascade, which is performed by Ub-activating enzyme E1, Ub -conjugating enzyme E2 and Ub-ligase E3. Depending on the number of ubiquitin molecules that are added to a specific protein, ubiquitination can be grouped into mono-ubiquitination and poly-ubiquitination, which are modified by a ubiquitin molecule and a ubiquitin chain, respectively 20. Mono-ubiquitination generally marks the substrate protein for a particular function, whereas polyubiquitinated substrate proteins are often degraded by the 26S proteasome. Therefore, histone ubiquitination largely occurs in the form of mono-ubiquitination. Like other histone modifications, ubiquitination is reversible, and the Ub marks can be removed by deubiqutinating enzymes. So far, 5%-15% of total H2A and 1%-2% H2B have been reported to be ubiquitinated. In contrast, H3 and H4 ubiquitination are less abundant. Histone ubiquitination could have distinct effects on transcriptional activity. For instance, H2B ubiquitination mediated by Rad6, a E2 enzyme, is linked to gene silencing 21, whereas ubiquitylation at lysine 123 of histone H2B contributes to gene activation 22. Furthermore, histone deubiquitination regulates transcriptional activity too. USP38 specifically removes the monoubiquitin of lysine 120 of H2B at lysine 120, which contributes to expression of proinflammatory cytokines Il6 and Il23a during lipopolysaccharides (LPS) stimulation by recruitment of demethylase KDM5B to the promoters of these genes 23, indicating that crosstalk among different histone modifications impacts on regulating transcriptional activity. In fact, accumulating evidence supports the idea that histone ubiquitination impacts transcriptional activity largely by its impact on other histone modifications.

### Epigenetic modulations associated with DNA

DNA methylation is the most studied epigenetic modification in mammals, in which methyl groups are added to the 5′position of a cytosine molecule without impacting on the DNA sequence. Methylation commonly occurs on the cytosine of CpG sites. Mammalian genomes exhibit high CpG methylation levels within gene regulatory regions, therefore methylation of CpG islands is the essential mechanism for regulation of gene expression.

DNA methylation includes de novo methylation and maintenance methylation. So far two distinct DNA methylation enzymes have been discovered, i.e., DNA methyltransferase 3a (DNMT3a) and DNMT3b, both of which contain a relatively conserved DNMT domain in the carboxy terminus and the chromatin reading domains including ATRX-DNMT3-DNMT3L and PWWP. Despite DNMT3a and DNMT3b share structural similarities, some studies showed that these two enzymes play different functions in performing de novo DNA methylation across the genome 24. In addition, DNMT1 has been reported to be involved in DNA methylation 25. However, the major activity of DNMT1 is considered to maintain the DNA methylation during DNA replication to prevent loss of methylation print on the parental strands 26. Methylated DNA will be demethylated by the TET methylcytosine dioxygenases that gradually oxidize 5mC to 5-hydroxymethylcytosine (5hmC), 5-formylcytosine (5fC) and 5-carboxylcytosine (5caC). The oxidization then promotes DNA demethylation during replication. In addition, certain demethylations like 5fC and 5caC, can happen through base removal by thymine DNA glycosylase during the base excision repair pathway. The dynamic regulation of DNA methylation process by the different enzymes controls gene expression, thereby impacting on the differentiation and function of various cells and organ development and maintenance.

### Epigenetic modulations associated with RNA

At RNA level, methylation is also universally found, accounting for more than 50% epigenetic modification in RNA, which includes 6-methyladenosine (m^6^A), 5-methylcytosine (m5C) and N1-methyladenosine (m1A). Similar to DNA methylation, RNA methylation is reversible, a process controlled by three regulators: writers, readers and erasers. RNA methylation is carried by “writers” (methyltransferases), which includes methyltransferase 3 (METTL3), METTL14 and METTL16 as well as protein subunit like Wilms' tumor 1-associating protein (WTAP) and Vir-like m^6^A methyltransferase associated (VIRMA) 27. These methylated groups at RNA can be removed by “erasers” (demethylases) including the fat mass and obesity associated (FTO) and alpha-ketoglutarate-dependent dioxygenase AlkB homolog 5 (ALKBH5). The dynamics of reversible RNA methylation is involved in multiple stage of nuclear RNA maturation, including mRNA metabolism, mRNA nuclear export, mRNA translation and mRNA stability. Another important regulator for RNA methylation is “readers” (binding proteins) which bind to methylation sites at RNA. The readers are direct and indirect types according to their ability to directly and specifically bind methylation sites. Direct readers contain five different YTH-containing proteins classified into three categories: YTH N6-Methyladenosine RNA Binding Protein C1/2 (YTHDC1/ YTHDC2) and YTH N6-Methyladenosine RNA Binding Protein F1/2/3 (YTHDF1, YTHDF2 and YTHDF3)^28^. In contrast, heterogeneous nuclear ribonucleoproteins (hnRNPs) are major indirect readers, which include hnRNPC, hnRNPG, and hnRNPA2/B1. In addition, the insulin-like growth factor 2 mRNA-binding proteins (IGF2BPs) also recognize the methylation sites at RNA. The readers determine the fate of target mRNAs by identifying methylation sites on distinct RNA transcripts.

In addition, non-coding RNAs (ncRNAs) have been widely considered as a type of epigenetic regulator. On the basis of size, non-coding RNAs can be grouped into short chain non-coding RNAs and long non-coding RNA (lncRNAs). Furthermore, short chain non-coding RNAs include siRNAs, miRNAs and piRNAs. LncRNAs can usually be classified into five categories: Sense, Antisense, Bidirectional, Intronic, and Intergenic lncRNA 29. Although ncRNAs are not translated into protein, they can regulate gene expression by different mechanisms. The major function of ncRNAs constitutes mediating transcriptional gene silencing by specifically interacting with their target sequences. In some cases, certain ncRNAs can interact with other epigenetic modulations including DNA methylation and histone modifications to alter gene expression.

## Epigenetics of immune cell development

**Figure 2:**
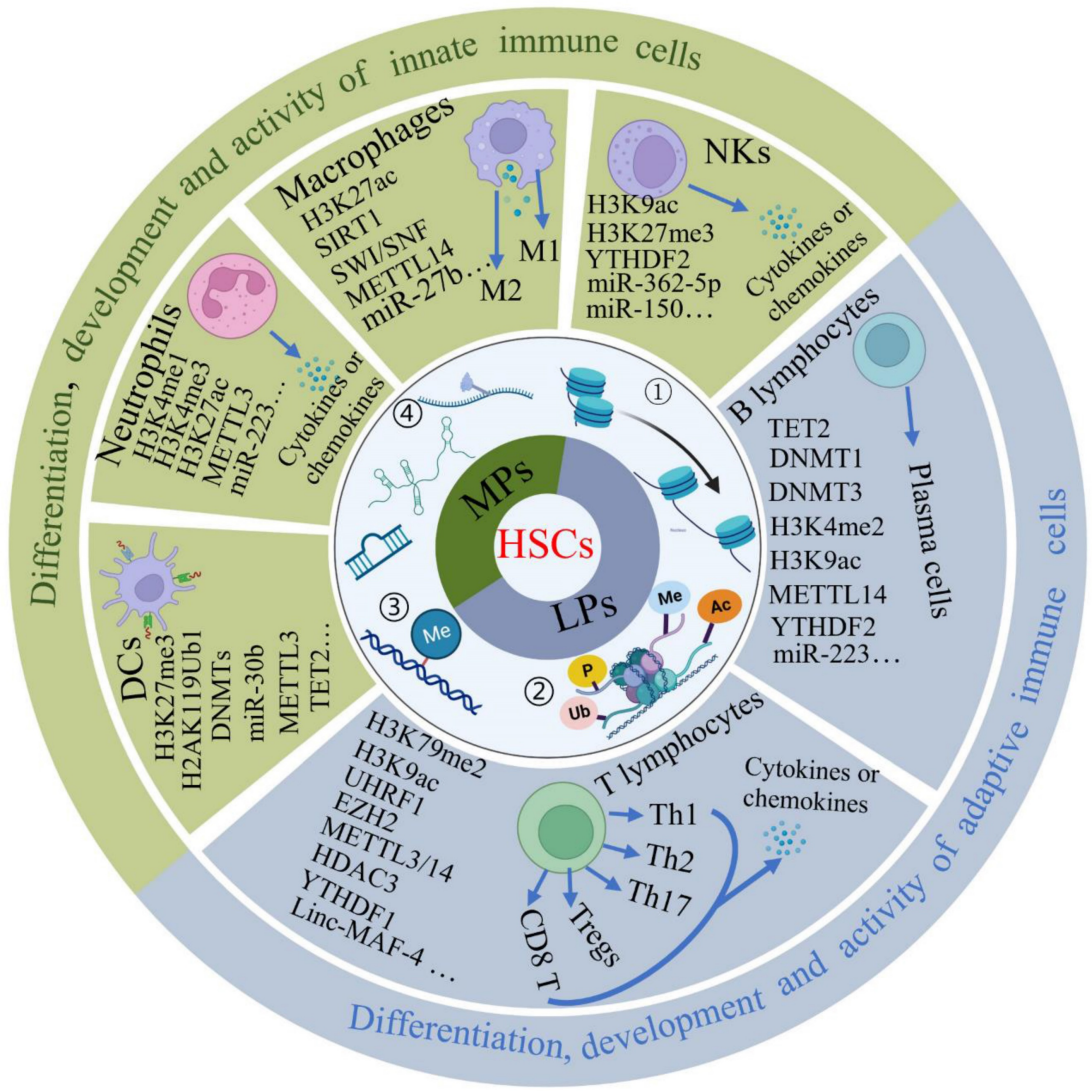
** The role of epigenetic modifications in the differentiation, development and activity of immune cells.** Hematopoietic stem cells (HSCs) produce all blood cell types including myeloid progenitors (MPs) and lymphoid progenitors (LPs). Here we majorly introduce three myeloid cells include macrophages, neutrophils and dendritic cells and lymphoid cells include T cells, B cells and natural killer cells. Four epigenetic modifications are indicated for differentiation, development and activity of immune cells. NKs: natural killer cells, DCs: dendritic cells, M1: M1-like macrophages, M2: M2-like macrophages, Th: T helper. ①-④ represent different type of epigenetic modifications.

The different cells of the immune system originate from the bone marrow, where hematopoietic stem cells (HSCs) can develop into all types of blood cells including myeloid-lineage and lymphoid-lineage cells, eventually comprising innate and adaptive immunity. Although both hematopoietic stem cells and mature cells share the same genome, their gene expression is dynamically regulated by epigenetic modulations including DNA methylation and histone modifications, enabling each type of immune cell to acquire distinct forms and functions. Here, we describe the regulatory effects of epigenetic modifications on the differentiation, development and function of T cells, B cells, natural killer cells (NKs), macrophages, neutrophils and dendritic cells (DCs) (Figure 2).

### Epigenetic modulations in T cell development

T cells comprise CD4^+^ and CD8^+^ T cells, which are defined on the basis of the expression of a different glycoprotein on their membrane surface. Both cell types are important in adaptive immunity. CD4^+^ T cells can differentiate into different subsets including Th1, Th2, Th17 and regulatory T cells (Tregs), which displays different effects on regulations of immune system. The precise regulation of these subsets is extremely important for immune homeostasis, and their dysfunction will lead to various human diseases.

Naïve T cells undergo various developmental stages to differentiate into different subsets, which are regulated by lineage specific TFs. When different subsets are matured, they can produce lineage specific cytokines and chemokines to act distinct functions. Growing evidence suggests that epigenetic modifications regulate T cells differentiation by targeting at lineage-specific genetic loci of TFs and effector cytokines. For instance, Scheer et al*.* reported that the lysine methyltransferase DOT1L-dependent dimethylation of lysine 79 of histone H3 (H3K79me2) regulates CD4^+^ Th cell differentiation by limiting Th1 lineage-specific gene expression 30. Accordingly, loss of DOT1L increases expression of Th1-specific genes and overproduction of IFN-γ, which suppresses Th2 cell differentiation, supporting the idea that IFN-γ expression contributes to Th1 differentiation developing from naive CD4^+^ T cells. Similarly, the histone H4 acetylation at the IFN-γ promoter of CD4^+^ T cells promotes Th1 differentiation by enhancing IFN-γ expression 31. Furthermore, increased accessibility of nucleosomal DNA at the IFN-γ promoter by recruitment of SWI/SNF complexes increases IFN-γ expression, allowing Th1 differentiation 32. The deficiency of METTL3, an m^6^A “writer” protein, in mouse T cells disrupts T cell homeostasis and differentiation 33. Mechanistically, METTL3-deficient naive T cells repress the mRNA decay of suppressors of cytokine signaling (SOCS) family genes encoding the STAT signaling inhibitory proteins including SOCS1 and SOCS3. The increased SOCS family activity inhibits IL-7-mediated STAT5 activation, thus preventing T cell homeostatic proliferation and differentiation. Interestingly, METTL3-deficient naive T cells also display the absence of METTL14 in T cells, indicating the similar function of both METTL3 and METTL14 in T cell. Indeed, METTL14 deficiency in T cells has been reported to impair naïve T cells differentiation. Furthermore, METTL14-deficient naive T cells show unregulated expression of Th1 cells-related cytokines including IFNγ and TNFα 34. In contrast, the level of Th2-potentiating cytokine IL25 is inhibited, indicating that T cell-specific METTL14 helps regulate function of Th1 and Th2. However, the detailed mechanisms need to be studied further.

During the period of Th2 cell differentiation, Th1-associated genes are inhibited through epigenetics. For instance, the expression of IFN-γ is repressed through the deposition of H3K27me3 to the IFN-γ gene locus by EZH2^35^. Moreover, Allan et al. reported that the deposition of H3K9me3 can recruit the heterochromatin protein 1α and promote transcriptional silencing at the promoter of Th1-specific genes by the formation of heterochromatin, allowing Th2 lineage stability 36. Similarly, Menin, a component of the TrxG complex, promotes differentiation of Th2 cells by reinforcing the levels of H3K9ac and H3K4me3 at the upstream regions of the GATA3 that enhances expression of Th2 cell-specific cytokines like IL-4and IL-13^37^. Intriguingly, GATA3 itself can regulate both active (H3K4me1 and 2) and repressive (H3K27me3) histone modifications of many target genes including Th2 cell-specific at their regulatory elements near GATA3 binding sites in a cell type-specific manner, thus regulating the Th2 cells differentiation 38. The permissive marks H3K9ac and H3K4me3 are also increased at loci of IL-4 gene, an essential gene for Th2 cell differentiation, which promotes Th2 cells differentiation by increasing IL-4 expression. Furthermore, the chromatin remodeling happens at most of the Th2 cytokine gene loci, indicating the important role played by epigenetics in Th2 cell differentiation. These studies support the idea that the expression of different cytokines and TFs due to epigenetic mechanisms determines mutually exclusive Th1 and Th2 cell differentiation.

Similarly, Th17 and Tregs differentiation are also mutually exclusive. Several critical genes like IL-6 are reported for Th17 differentiation from naive T cells by inducing the STAT3 signaling pathway, which is responsible for the inhibition of Tregs differentiation 39. Therefore, the epigenetic modifications of these TFs and cytokines impact their fates. For instance, Lin et al. found that Cxxc finger protein 1 (Cxxc1) epigenetically promotes the generation of Th17^40^. Mechanistically, Cxxc1 binds to IL6Rα gene loci by maintaining the appropriate H3K4me3 modification of its promoter, which controls the IL-6/STAT3 signaling pathway, preventing their differentiation into Tregs. Likewise, IL-6R deficiency disrupts IL-6/STAT3 signaling, which stimulates the production of Tregs instead of Th17 cells, suggesting that Cxxc1 epigenetically controls a balance between Th17 and Tregs via the IL-6/STAT3 signaling pathway. Furthermore, STAT3 binding to the IL-17 promoter is associated with an elevation of the H3K4me3 at the IL-17 loci, which is therefore necessary for the expression of Th17-specific cytokines IL-17 and IL-21^41^. In addition, ATF7ip, an epigenetic regulator for H3K9me3 expression, as a repressor of IL2 gene expression through H3K9me3 deposition in the IL2-IL21 intergenic region, plays an important role in Th17 differentiation 42.

DNA methylation is an important epigenetic mechanism for regulating Tregs differentiation. Compared to undifferentiated CD4^+^ T cells, the globally hypomethylated DNA of Tregs has been reported to regulate Tregs-related gene expression like FOXP3 and CTLA4^43^. It has been shown that conserved noncoding sequence 2 (CNS2), a CpG-rich Foxp3 intronic cis-element, specifically demethylates during Tregs differentiation 44. CNS2 protects Foxp3 expression from destabilizing cytokine conditions by sensing TCR/NFAT activation, which promotes the interaction between CNS2 and Foxp3 promoter 44. 5-Azacytidine (5-Aza), a DNA methyltransferase inhibitor, has been reported to promote Tregs signature gene expression including FOXP3 and CTLA-4. Further analysis showed that 5-Aza treatment leads to the overproduction of IL-2, which is necessary for maintenance of FOXP3 expression 45. Helmin et al. showed that epigenetic regulator UHRF1 is essential for maintenance of methyl-DNA marks that stabilize Tregs cellular identity, and deletion of UHRF1 causes a widespread decrease in CpG methylation, causing destabilization of the Tregs lineage 46. These findings suggest that DNA methylation plays an important role in regulating Tregs differentiation from naïve T cells. Emerging evidence has shown that other epigenetic mechanisms are involved in Tregs differentiation and development. For instance, Bmi1 deficiency results in loss of Tregs identity and Tregs are converted into Th1/Th17-like cells because of upregulation of H3K27ac and chromatin accessibility 47. Furthermore, METTL3 and METTL14 deficiency impairs the differentiation of naïve T cells into induced Tregs and the suppressive activity of Tregs 34,48. Whether there is crosstalk among different epigenetic modifications warrants further study.

Likewise, CD8^+^ T cell differentiation is also regulated by different epigenetic mechanisms. The comparison of the epigenetic landscapes between naïve CD8^+^ T cells, memory and effector CD8^+^ T cells reveals that both enhancers and promoters are important for CD8^+^ T cell differentiation 49. Furthermore, EZH2 is considered an important determinant for differentiation of effector CD8^+^ T cells but not for memory CD8^+^ T cell differentiation by regulating H3K27me3 deposition on pro-memory CD8^+^ T cell associated genes 50. Another study reported that DNMT3a determines the development of memory CD8^+^ T precursor cells and allows terminal effector CD8^+^ T cell differentiation 51. Loss of DNMT3a biases the differentiation of early CD8^+^ T effector cells into memory CD8^+^ T precursor cells. On the other hand, T cell-specific knockout of DOT1L causes loss of H3K79me2 in T cells, which results in loss of naïve CD8^+^ T cells and premature differentiation toward a memory-like state 52. Importantly, epigenetic mechanisms are also involved in regulation of CD8^+^ T cell activity. Tay et al. demonstrated that HDAC3 suppresses gene expression associated with cytotoxicity during activation and is required for persistence of activated CD8^+^ T cells 53. Therefore, inactivation of the HDAC3 strongly augments the cytotoxic function of CD8^+^ T cells by increasing cytotoxic cytokines like granzyme B (GzmB) and IFN-γ. These studies indicate that epigenetic regulators are important determinants for CD8^+^ T cell differentiation, development and function.

### Epigenetic modulations in B cell development

B cells are responsible primarily for the basic functions of antibody production. Their abnormal differentiation, maturation and function are connected to several human diseases. Numerous studies have showed that epigenetic modifications are involved in the processes of B cell development, differentiation and function. For instance, naïve B cell show an inactive epigenetic status including genome-wide DNA hypermethylation and histone deacetylation 54. After antigen stimuli, naïve B cells divide and then differentiate into germinal center (GC) B cells, which further differentiate into either plasma or memory B cells. During this period, the status of genome-wide DNA changes from hypermethylation to hypomethylation, with concordant inverse changes in gene expression affecting most notably genes of the NF-kB and MAPK signaling pathways, indicating that these pathways are important for B cells differentiation 54. Consistently, NF-kB has been reported to maintain B cell differentiation and function, e.g. binding the enhancer of the immunoglobulin κ light-chain gene 55. Indeed, Orlanski et al. demonstrated that TET2/TET3 conditional knockout at early stages of B-cell development largely inhibits lineage-specific programmed demethylation events, which affects the expression of B cells lineage genes by impairing enhancer activity, thus causing defects in B cell differentiation and function 56. Similarly, deletion of TET2 and TET3 promotes GC B cells responses, while knockout of DNMT1 in TET-deficient B cells abrogates this effect, consistent with the opposing functions of DNMT and TET enzymes in regulating DNA methylation and demethylation 57. RNA m^6^A modifications have been involved in B cell development. B cell-specific deletion of METTL14 blocks two key transitions in B cell development including pro-B cell proliferation and the large-pre-B-to-small-pre-B transition. In contrast, YTHDF2 is only critical for pro-B cell proliferation 58. Similarly, METTL14 promotes mRNA decay of negative immune regulators like Lax1 and Tipe2, thus enhances expression of the gene required for GC B cell positive selection and proliferation. Therefore, ablation of METTL14 in B cells results in compromised GC B cell proliferation and a defective antibody response in mice, indicating that METTL14 is essential for the GC B cell response 59. In addition, Zhang et al. reported that deletion of KMT2D, a H3K4 methyltransferase, induces a global reduction in H3K4me1, H3K4me2 and H3K4me3, which results in an increase in GC B cells and enhances B cell proliferation by upregulating BCL2 expression 60. In contrast, deletion of LSD1, a histone demethylase, reduces cell proliferation and differentiation of GC B cells due to decreased demethylation of H3K4me1 and H3K4me2^61^, indicating the important role of histone methylation in B cell development.

Plasma cells represent the terminal differentiation step of mature B cells, which are regulated by epigenetic modifications. Deficiency of DNMT3 increases expansion of plasma cell populations upon immunization, indicating that DNA methylation is involved in maturation of plasma cells 62. Furthermore, gene expression is mostly unaltered in naïve and GC B cells but is prominently dysregulated in DNMT3-deficient plasma cells, coinciding with the increased chromatin accessibility at E2A, PU.1 and IRF4 binding motifs in plasma cells. These findings suggest that de novo DNA methylation by DNMT3 represses the plasma cell chromatin state and regulates plasma cell differentiation by suppressing the gene expression of key activation-related genes. In contrast, conditional deletion of TET2 and TET3 in B cells inhibits differentiation of plasma cells, partially because TET2- and TET3-dependent demethylation is essential for sustaining high IRF4 expression, which has an important role in plasma cell differentiation 63. Qi et al. also demonstrated that ascorbic acid promotes plasma cells differentiation by enhancing the expression of several TFs like IRF4^64^. Further analysis demonstrated that the effect of ascorbic acid on plasma cell differentiation is dependent on TET2/3-mediated DNA demethylation, as ascorbic acid stimulation does not increase expression levels of key TFs involved in plasma cell differentiation including IRF4 and PRDM in TET2 and TET3 double knockout B cells, supporting an important effect of DNA demethylation on plasma B cell maturation.

For memory B cell differentiation, epigenetic modifications have been widely reported too. For instance, the memory B cell differentiation related genes like CD27 seem to be controlled by histone modifications 65. In Cγ1-Cre EZH2^fl/fl^ mice, the EZH2 deficiency in the B cell differentiation stage leads to impairment of memory B cell formation likely due to suppression of PRDM1 and IRF4 transcription by EZH2, suggesting that EZH2 is essential for generation of memory B cells 66. One study showed that miR-15a and miR-16 have been reported to regulate memory B cells survival by targeting BCL2^67^. Zhang et al. also demonstrated that miR-223 is enriched in human memory B cells and is involved in B cell differentiation by downregulating the expression of LMO2 and BLIMP1^68^. Furthermore, multiple lncRNAs have been shown to be preferentially expressed in human memory B cells, but not in naïve B cells 69, suggesting that lncRNA plays an important role in memory B cell differentiation. Therefore, different types of epigenetic mechanisms regulate specific gene expression, which results in B cell differentiation into plasma cells and memory B cell, providing potential targets for these B cell dysfunction related diseases.

### Epigenetic modulations in NKs development

NKs are the first line of defense against infectious agents and cancer cells by secreting multiple chemokines (CCL3, CCL4, CCL5, and XCL1), cytokines (IFN-γ, TGF-β, and IL-10) and growth factors (GM-CSF). During the terminal differentiation process, NKs gradually obtain the capability to produce IFN-γ through demethylation and epigenetic remodeling at the IFNG promoter 70. In contrast, methylation of the promoter decreased NKs IFNG transcriptional activity in NKs. Furthermore, DNA methylation has been indicated in regulating gene expression of various NKs receptors including killer Ig-like receptors (KIRs) and natural cytotoxic receptors (NCRs), thus influencing their killing ability. Indeed, methylation of the promoter of KIR genes consistently suppresses KIR expression, leading to inhibition of NKs activity due to decreased GzmB and perforin release. In contrast, when NKs begin to differentiate, KIR genes are demethylated causing their transcription. Accordingly, Gao et al. depicted that the opening of the chromatin structure occurs before the process of DNA demethylation 71. The chromatin stays open for expression of KIR genes, whereas non-expressed genes are located in condensed chromatin structures, indicating that chromatin accessibility is an early event involved in the expression of KIR genes in NKs. In addition, chromatin regulation has been shown to regulate NKs development by interfering with other genes. For instance, MYSM1, a histone H2A deubiquitinase, promotes NKs development by maintaining an active chromatin at the ID2 locus 72. A significantly high level of AcH3, especially H3K9ac, is observed in the NKG2D (an NCRs related gene) in NKs, whereas repressive histone modifications (H3K27me3 and H3K9me2) of the NKG2D in NKs are hardly detectable 73, indicating that histone modifications are involved in regulating NKs cytotoxicity.

Based on the miRNA transcriptomes from NKs, Ni et. al revealed differential miRNA expression analysis in various human NKs populations 74. Of note, miR-362-5p, a novel miRNA, is identified as a regulator in NKs by targeting CYLD, a negative regulator of NF-κB signaling. Therefore, overexpression of miR-362-5p can promote the function of human primary NKs by enhancing the expression of IFN-γ, perforin and GzmB, the major effector molecules of NKs. The miR-27a* has been reported to specifically bind to the 3' untranslated regions of PRF1 and GzmB, which results in decreased expression of perforin and GzmB in both resting and activated NKs, thus inhibiting NKs cytotoxicity 75. Similarly, PRF1 could be targeted by miR-30e 76 and miR-150 77. Therefore, these microRNAs also regulate NKs cytotoxicity.

Meanwhile, RNA modifications are important for NK activity. When METTL3 is deleted in NK cells, the expression of SHP2, a tyrosine phosphatase, is decreased, which represses the activation of the AKT and MAPK signaling pathway, thus leading to NK cells hyporesponsive to IL-15 and accelerated tumor development and shortened survival in mice 78. Similarly, YTHDF2 deficiency in NK cells impairs NK cell antitumor and antiviral activity by repressing mRNA degradation of TARDBP, which is an important cell-cycle negative regulator during cell division 79. These data suggest that RNA-associated epigenetic mechanisms play an important role in NKs activity.

### Epigenetic modulations in macrophage development

Macrophages are differentiated monocytes and an important type of innate immune cell. Activated macrophages can be divided into two kinds: M1-like macrophages (M1) and M2-like macrophages (M2). The differentiation process from monocytes to macrophages is accompanied by epigenetic modulations including DNA methylation. Differential DNA methylation is typically restricted to CpG sites that are characteristic of increased binding of TFs like C/EBP and ETS, which are known to be involved in monocyte-to-macrophage differentiation. Wallner et al. further reported that DNA demethylation can mediate the structure and function of macrophages by regulating a small number of classical genes that are involved in the actin cytoskeleton and phagocytosis 80.

In the whole genome, IFN-γ priming induces approximately 5,000 new H3K27ac peaks in distal and intergenic regions 81. The additive stimulation of IFN-γ priming macrophages by LPS, a macrophage activator, further increases an additional 6,000 unique H3K27ac peaks in the genome, indicating that IFN-γ alters the regulatory and enhancer landscape to modulate macrophage responses to inflammatory stimulation. Interestingly, the epigenetic mechanisms could be different between M1 and M2 macrophage differentiation. For instance, JMJD3-mediated H3K27 demethylation is crucial for mediating M2 macrophage development, but is dispensable for M1 responses 82. This could be associated with different marker genes for M1 and M2. M1 modulates inflammatory responses whereas M2 is mainly involved in anti-inflammatory responses. Indeed, TFs such as STAT6 are involved in polarization of M2 macrophages 83. However, the different epigenetic landscapes between M1 and M2 macrophages warrant further study.

RNA-associated epigenetic modifications have been indicated in macrophage development. Based on an RNA binding protein focused CRISPR screening, Tong et al. found that several m^6^A writers are the top candidate genes regulating macrophage activation by LPS. Further analysis demonstrated that loss of METTL3 causes upregulated expression of IRAKM by inhibiting m^6^A modification on IRAKM mRNA and slowing down its degradation, which eventually suppresses TLR signaling-mediated macrophage activation 84. Furthermore, the migration of macrophage could be regulated by METTL3. The depletion of METTL3 in monocyte-derived macrophages enhances macrophages migration by decreasing expression of tubulin acetyltransferase 1 and reducing acetylation of α-tubulin 85. YTHDF2 has been reported as a negative regulator for macrophage development. Its deficiency enhances expression of LPS-induced inflammatory cytokines including TNF-α, IL-6, IL-1β and IL-12 by stabilizing the mRNA of MAP2K4 and MAP4K4 86. In addition, many microRNAs have been shown to regulate macrophage differentiation and activation. For instance, high levels of miR-9 contribute to differentiation of monocytes into M1 by regulating PPARδ expression. Similarly, the expression of miR-27b can be increased by LPS stimulus 87. Furthermore, inhibition of miR-27b impairs the ability of LPS to reduce the PPARγ mRNA half-life, indicating that miR-27b influences M1 activation by modulating PPARγ expression. These studies support the important role of RNA modifications in macrophage development and function.

In addition, enhancers show extensive enrichment for motifs for the binding of TFs including C/EBP, PU.1, IRF and AP-1, all of which are necessary for the function and development of macrophages 88. In contrast, expression of SIRT1 decreased in macrophages stimulated by LPS 89. Myeloid-specific SIRT1 deletion suppresses LPS-induced IRF8 expression. Mechanistically, SIRT1 interacts with IRF8 and increases its expression by inhibiting the acetylation level of IRF8. DNMT3a, a DNA methyltransferase, has been reported to increase IFN-β production by maintaining a high expression of HDAC9 in naive peritoneal macrophages, promoting their activity 90. Another study revealed that during macrophage development, DNA repair enzymes like BRCA1 and PARP1, are activated by the SWI/SNF chromatin remodeling complex that works as the histone acetylation sensor to regulate EP300 and HDAC1 activities 91. In turn, the EP300-HDAC-SWI/SNF axis controls the chromatin structure and transcriptional activity of DNA repair enzyme promoters in the monocyte-macrophage differentiation axis.

### Epigenetic modulations in neutrophil development

Neutrophils are an important and abundant effector in the innate arm of the immune system. However, growing evidence shows that the function of neutrophils is deficient during the first weeks of life, including the expression of cytokine genes such as IFNγ, indicating that postnatal epigenetic modulations play an important role in development of neutrophils. Indeed, several epigenetic TFs like PU.1, C/EBPα and C/EBPβ are universally expressed at high levels throughout neutrophil development 92. These TFs are also required for normal neutrophil differentiation so that their abnormal expression is connected to neutropenia. For instance, C/EBPε is expressed by lineage-committed granulocyte progenitors, and its deficiency causes neutrophil progenitor arrest and neutropenia 93.

Recently, Liu et al. reported that ALKBH5-deficient neutrophils exhibit impaired migration ability by increasing the expression of neutrophil migration-related molecules CXCR2 and NLRP12, and reducing expression of neutrophil migration-suppressive PTGER4, TNC and WNK1^94^, indicating that ALKBH5 plays an important role in regulating neutrophil migration. This notion is further supported by the study that ALKBH5-deficient mice show high retention of mature neutrophils in bone marrow and defective neutrophil release into blood. Mechanistically, loss of ALKBH5 erases m^6^A methylation on CSF3R mRNAs to suppress their decay, thus upregulating the cell-surface G-CSFR expression and JAK-STAT signalling 95. Another study also reported that METTL3 modulates neutrophil release from bone marrow to bloodstream by enhanced translation and decreased degradation of mRNA of a Toll-like receptor 4 96. Interestingly, METTL3 has been found to control neutrophil extracellular traps-mediated ferroptosis by targeting glutathione peroxidase 4, an important regulator in the ferroptosis process 97. In addition, miR-223 plays an important role in regulating neutrophil function by suppressing the IL-6-NCF-2 pathway 98. These studies confirm the important role of RNA-associated epigenetic regulators in neutrophil function.

In fact, the interindividual variability in DNA methylation profiles is widely found in human neutrophils. During normal neutrophil development, DNA methylation undergoes a dynamic change. Furthermore, gene body and upstream regions show higher variation in DNA methylation compared to gene promoters. Another study demonstrated that neutrophil-specific DNA methylation hypervariable sites are enriched at dynamic chromatin regions and active enhancers 99, indicating that epigenomic mechanisms may fine-tune neutrophil gene expression. Grassi et al. further described the epigenetic landscape by profiling four active histone marks (H3K4me3, H3K27ac, H3K4me1 and H3K36me3) and two repressive marks (H3K9me3 and H3K27me3) as well as the DNA methylation status in the five differentiation stages from bone marrow-residing progenitors to mature neutrophils cells 92. Different stages are the successive transitions with the largest numbers of differentially expressed genes and consecutive regulation in chromatin statuses. Overall, these transcriptomic and epigenomic differences reflect the distinct expression of genes responsible for the functional disparities among these stages of neutrophil development.

### Epigenetic modulations in DCs development

DCs represent a subset of innate immune cells that play a crucial role in anti-tumor responses by sampling and presenting tumor-related antigens to T cells. Their maturation and function are dependent on the expression of certain genes like major histocompatibility complex (MHC) and GM-CSF, whose expression could be regulated by epigenetic modulations. Not surprisingly, epigenetic modulations like DNA methylation and histone modifications have been indicated in the development of DCs. The first DNA methylation map of DCs differentiation and maturation shows that DNA methylation changes happen at non-CpG island and TFs binding sites, along with the expression of TET2 and DNMTs 100. These DNA methylation regulators control the DCs development related pathways like GM-CSF pathway, thus affecting DCs development. TET2-dependent demethylation is also essential for acquiring proper dendritic cell identity by increasing DC-specific gene expression 101, indicating the important role of the balance between DNA methylation and demethylation in determining DCs development.

RNA-associated epigenetic mechanisms have been indicated in DCs development. Su et al. performed miRNA profile of HSCs, immature and mature DCs and found that 391 miRNAs are differentially expressed during DCs differentiation 102. Interestingly, the overlap of miRNA expression between each developmental stage is observed. For instance, miR-132 and miR-147 are highly expressed in immature and mature DCs and their high expression are not observed in HSCs, indicating their critical role in maintaining DCs-lineage identity. Furthermore, several other microRNAs like miR-511-3p, miR-30b and miR-544 have been reported to function as important roles in DCs differentiation 103,104. Recently, METTL3-mediated m^6^A modification has been widely reported to modulate DCs activation and maturation. Wang et al., reported that METTL3 deficiency in DCs impairs phenotypic and functional maturation of DCs, reducing the ability to stimulate T cell responses both *in vitro* and *in vivo*. Mechanistically, METTL3-mediated m^6^A of CD40, CD80 and TLR4 signaling adaptor TIRAP transcripts augments their translation into DCs for stimulating T cell activation, and strengthening TLR4/NF-κB signaling-induced secretion of inflammatory cytokine 105. In addition to inflammatory production, METTL3 is also involved in the expression of MHC-II and costimulatory molecules (CD80, CD86) in DCs, which play important roles in DCs induced immune tolerance. Therefore, loss of METTL3 in DCs induces immune tolerance and prolongs allograft survival in mouse heart transplantation 106. In addition, loss of YTHDF1 in classical DCs enhances the cross-presentation of tumor antigen and the cross-priming of CD8^+^ T cells in vivo by repressing translation of lysosomal proteases that can destruct internalized antigens 107.

Besides DNA- and RNA- associated epigenetic mechanisms, histone modifications are important in DCs development. For instance, Yi et al. reported that intracellular HSP70L1 could inhibit DCs maturation by promoting suppressive H3K27me3 and H2AK119Ub1 histone modifications at the promoter regions of the MHC and STAT3 genes 108. Another study showed that changes in H3K9ac levels largely correspond to changes in expression of multiple genes including FLT3, PU.1, TCF4, IRF8 and ID2, which are known as DCs differentiation related regulators 109. Therefore, HDAC inhibition impairs the establishment of a DCs-specific gene expression repertoire. In addition, FLT3 expression is also regulated by MYSM1, a histone H2A deubiquitinase, by impacting histone modifications at the FLT3 promoter region 109. Deletion of MYSM1 specifically impairs development of steady-state DCs, but not other myeloid lineages like monocyte and macrophage. Taken together, epigenetic regulators play an essential role in maintaining DCs development.

## Epigenetics of immune cells in tumor progression

**Figure 3:**
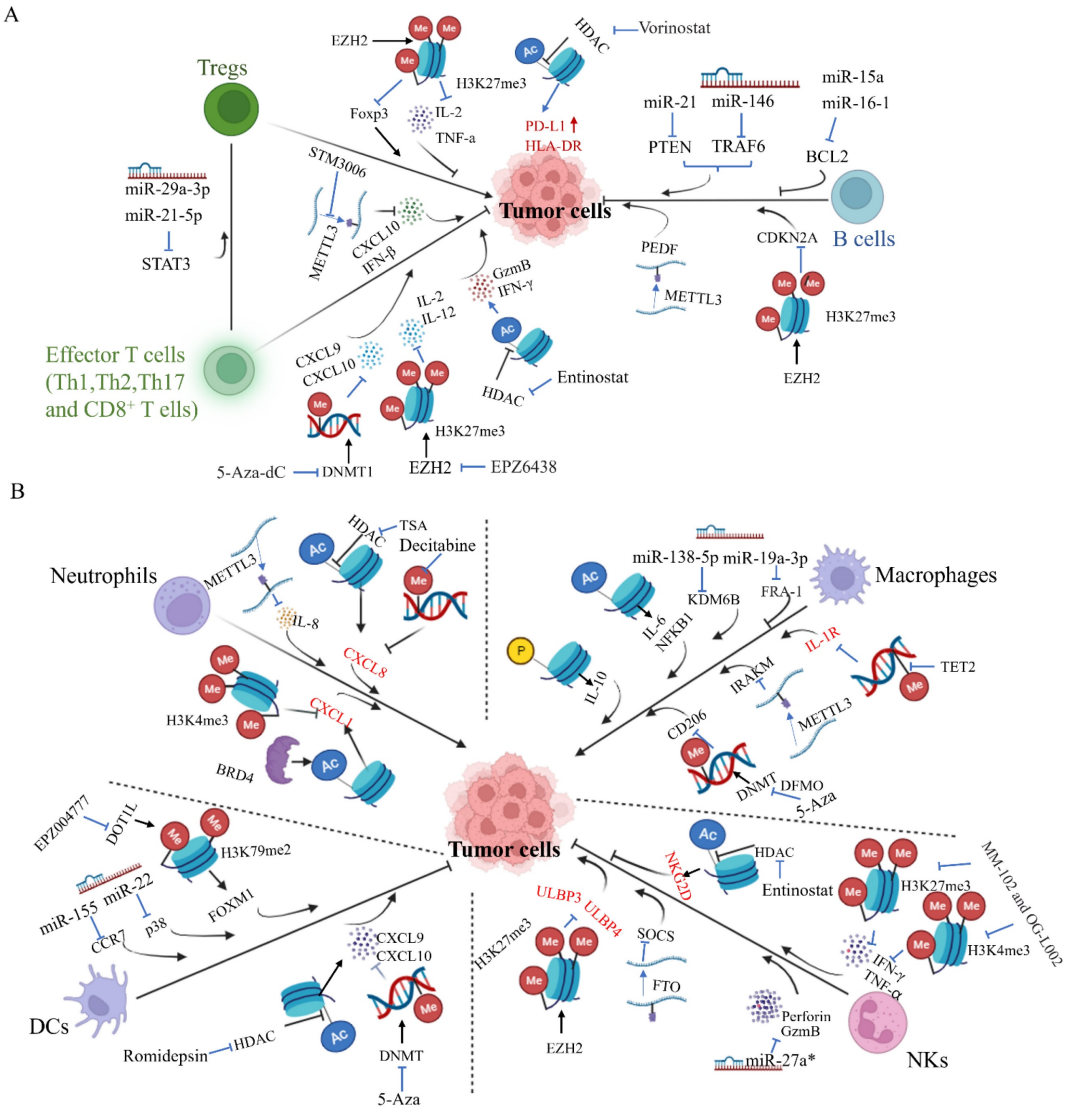
** The epigenetic modifications of immune cells in tumor microenvironment.** A. The epigenetic modifications of adaptive immune cells (B and T cells) in tumor progression. Different epigenetic modulators like histone methylation regulator H3K27me3 and DNA methylation regulator DNMT1 have been shown to regulate development and/ or activity of immune cells in distinct tumor progression. On the other hand, the effect of epigenetic modulation like histone deacetylation regulator HDAC on tumor cells could also regulate infiltration of immune cells into TME by regulating the expression of genes like PD-L1 and HLA-DR on tumor cells. B. The epigenetic modifications of innate immune cells (NKs, macrophages, neutrophils and DCs) in tumor progression. Different epigenetic modulators like histone methylation regulator H3K79me2 and microRNAs have been shown to regulate development and/ or activity of immune cells in distinct tumor t ypes. Meanwhile, many epigenetic regulators like DNA methylation regulator TET2 and miR-93, regulating IL-1R and ULBP3 expression in the tumor cells, respectively, which impact on tumor progression. The genes marked in red represent the effect of epigenetic modulations on expression of genes in tumor cells, while the genes marked in black represent the effect of epigenetic modulations on expression of genes in immune cells. In both cases, the expression of these genes interferes with tumor progression by immune cells. Arrows (↑) illustrate promoting and blunt ended lines (┬) dictate inhibiting effects.

It is well documented that tumor progression is accompanied by abnormalities of the immune system. In principle, tumor progression should be surveilled by cytotoxic innate and adaptive immune cells. However, as the tumor develops from neoplastic tissue to clinically detectable tumors, cancer cells evolve different mechanisms that mimic peripheral immune tolerance in order to escape immunosurveillance. Accumulating evidence suggests that epigenetic dysfunction of tumor cells or different immune cells is closely associated with immunosurveillance failure, leading to tumor initiation and progression (Figure 3).

### Epigenetics of T cells in tumor progression

The normal development and function of distinct T cells subsets are essential for immunological homeostasis. Tumorigenesis is often accompanied by dysfunction of the immune system, including increased Tregs and reduced cytotoxicity CD8^+^ T cells. It would not be surprising if epigenetic modulations of T cells are closely associated with tumor progression by influencing development and function of different T subsets. For instance, loss of METTL3 or METTL14 in tumor cells increased tumor-infiltrating CD8^+^ T cells and promoted secretion of IFN-γ and CXCL10 by stabilizing the STAT1 and IRF1 mRNA via YTHDF2, thus sensitizing microsatellite instability-low CRC tumors to PD-1 treatment in vivo 110. Similarly, Guirguis et al. reported that pharmacological inhibition of METTL3 by STM3006 augments CD8^+^ T cells function by inducing dsRNA-sensing and interferon signaling 111. More importantly, METTL3 inhibition shows similar efficacity compared to anti-PD1 therapy. Further analysis revealed that METTL3 inhibition and anti-PD1 therapy target distinct malignant clones. Unexpectedly, METTL3 and METTL14 showed dissimilar effects on tumor-infiltrating CD8^+^ T cell in the TME of breast cancer 112,113. Moreover, some miRNAs are reported to regulate tumor progression by influencing immune cell function. For example, Zhou et al. reported that miR-29a-3p and miR-21-5p synergistically induce the Tregs/Th17 cell imbalance through direct targeting of STAT3 in CD4^+^ T cells, which enhance the growth and metastasis of ovarian cancer cells 114.

In a human ovarian cancer model, H3K27me3 mediated by EZH2 at their promoter regions represses the tumor production of Th1-associated chemokines like CXCL10, which influences tumor progression by mediating recruitment of effector T cells to TME 115. Furthermore, pharmacological or genetic ablation of H3K27me3 augments Th1-associated chemokine expression. In ovarian cancer cells DNMT1-mediated DNA methylation causes aggressive phenotypes by suppressing the tumor production of Th1-associated chemokines CXCL10 115. In addition, inhibition of DNA methylation in tumor cells via 5-Aza-dC increases Th1 cells infiltration in the TME, which represses tumor progression and improves the therapeutic efficacy of PD-L1 blockade therapy. Consistently, Goswami et al. reported that inhibition of EZH2 increases infiltration of Th1 cells and cytotoxic effector T cells in the TME as well as a depletion of Tregs in bladder cancer murine model 116. Interestingly, they also demonstrated that Ipilimumab (an anti-CTLA4 antibody) increases EZH2 expression in CD4^+^ T cells, causing potential resistance to anti-CTLA4 therapy. Indeed, inhibition of EZH2 improves the response to anti-CTLA4 by regulating tumor cytotoxic effector T cells and changing the phenotype of Tregs into effector-like T cells. Furthermore, in mouse and human prostate cancer organoids models, inhibition of EZH2 also increases the expression of the Th1 attracting chemokines CXCL9 and CXCL10 as well as Th1 cytokines including IL-2 and IL-12, which enhances CD4^+^ and CD8^+^ T cells infiltration in the TME and sensitizes prostate cancer to anti-PD1 therapy 117. In addition, JQ1, a bromodomain-targeted BET inhibitor, increases Th1 cells as well as depletion of Tregs in the TME and improves survival in lung cancer mouse model 118. These studies imply that epigenetic regulation of Th1 cells is an important approach to cancer treatment. Indeed, increased Th1 cells were considered as a potential approach to repress bone metastasis in patients with castration-resistant prostate cancer 119.

In addition, the role of HDAC inhibitors as an immunomodulatory agent has been extensively studied. Zheng et al. reported that HDACs are involved in the repression of T cell-associated chemokines such as CXCL10 in tumor cells and T cells 120. Therefore, HDAC inhibitors diminish lung tumor growth by upregulating the expression of multiple T-cell chemokines in cancer cells and T cells as well as enhancing T-cell infiltration in the TME. Importantly, HDAC inhibitors significantly enhance the response to PD1 blockade therapy in multiple lung tumor models, indicating that the combination of HDAC inhibitors and PD1 blockade therapy represents a promising strategy for lung cancer treatment. Similarly, CXD101, a class 1 HDAC inhibitor, enhances CD4^+^ and CD8^+^ T-cell infiltration in the TME by affecting immune-relevant gene expression in human colorectal cancer (CRC) cell lines 121. Therefore, the combination of CXD101 and anti-PD1 or anti-CTLA4 treatment causes significant anti-tumor activity compared to anti-PD1 or anti-CTLA4 treatment alone in CRC mouse model. Interestingly, Belinostat, a HDAC inhibitor, improves the anti-tumor activity of anti-CTLA4 but not of anti-PD1 therapy in a murine hepatocellular carcinoma model 122. A greater relevance of CTLA4 and/or Tregs than PD1/PD-L1 as immunosuppressive mechanisms at initial tumor stages may explain the superior sensitivity to anti-CTLA4 in this model. Moreover, Trichostatin A (an HDAC inhibitor) inhibits apoptosis of CD4^+^ T cells in the melanoma TME by suppressing NFAT1-regulated FasL expression, and therefore its combination with anti-CTLA4 could enhance the infiltration of CD4^+^ T cells and promote anti-tumor effects of anti-CTLA4 123. Furthermore, entinostat, a class I HDAC inhibitor, has been reported to augment the efficacy of the anti-cancer vaccine. N-803 plus vaccine only induce limited tumor infiltration of CD8^+^ T cells with minimal levels of GzmB^124^. In contrast, the addition of entinostat to N-803 plus vaccine suppresses significant tumor growth, correlating with increased expression of genes associated with tumor inflammation, enhanced infiltration of activated CD8^+^ T cells with maximal GzmB as well as increased serum IFNγ in the TME of TNBC and CRC murine carcinoma models 124. Another study reported that HDAC inhibitors significantly enhance the in vivo response to PD1/CTLA4 blockade in TNBC mouse model. This effect is, at least in part, due to upregulation of PD-L1 and HLA-DR in tumor cells as well as the decrease of the recruitment of Tregs in the TME 125. Altogether, epigenetic therapies induce a favorable immune context in the TME that improves the response to immunotherapy by modulating T-cell polarization and recruitment.

### Epigenetics of B cells in tumor progression

A high frequency of somatic mutations in genes that encode epigenetic enzymes in B cells is observed in lymphomas 126, indicating the epigenetic dysfunction could be the reason for lymphomas. Indeed, the aberrant expression of DNMT3b and TET1 has been widely found in B-cell lymphomas 127,128. Whole-exome sequencing data reveals that TET1-deficient tumors cause increased self-renewal and DNA damage due to aberrant hypermethylation of DNA, contributing to the generation of pre-malignant B cells at the pro-B cell stage of differentiation. Similarly, TET2 deficiency results in DNA hypermethylation of regulatory elements in GC B cells via promoter hypermethylation and loss of enhancer 5hmC 129. This hypermethylation interferes with the binding of TFs including those involved in exit from the GC reaction and involves pathways such as B cells receptor, antigen presentation and CD40, thus contributing to lymphomagenesis.

Velichutina et al. reported that EZH2-bound promoters are aberrantly hypermethylated in diffuse large B-cell lymphoma cells (DLBCL), and knockdown of EZH2 in diffuse DLBCL resulted in acute cell cycle arrest at the G 1/S transition by increasing expression of several key cell cycle-related tumor suppressor genes, indicating that EZH2 expression is closely associated with B lymphomagenesis 130. Indeed, somatic mutations at Y641 and A677 residues within the catalytic domain of EZH2 have been widely found in DLBCL 131. These mutations change the enzymatic activity of EZH2, leading to a protein that fails to recognize unmodified H3K27 and preferentially converts mono- or dimethylated H3K27 to the trimethylated state 132, which results in the aberrant and permanent silencing of the cell cycle checkpoint and plasma B cell differentiation genes that EZH2 represses. Not surprisingly, EZH2 inhibitors can induce proliferation arrest and apoptosis in DLBCL cells with EZH2 mutations 133. Brach et al. also showed that tazemetostat, a EZH2 inhibitor, causes inhibition of tumor growth in both EZH2-mutant (Y646F) and EZH2- DLBCL xenografts due to a loss of H3K27 trimethylation 134. Furthermore, EPZ011989, a selective and orally available EZH2 inhibitor, significantly delays tumor growth in a mouse xenograft model of human B cells lymphoma, supporting the important role of EZH2 in DLBCL 135. In addition, many other histone modification related enzymes, such as CBP, MLL2, UTX and JMJD2C, are frequently mutated in B cells lymphomas, indicating their important roles in carcinogenesis by affecting B cells.

Abnormal RNA-associated epigenetic mechanisms also cause B lymphomagenesis. For instance, miR-15a and miR-16-1 negatively regulate BCL2 at a post-transcriptional level, and overexpression of these miRNAs induces apoptosis by repressing BCL2 expression in chronic lymphocytic leukemia B cells 136. Overexpression of miR-181a results in G0/G1 cell cycle arrest and increased apoptosis by repressing CARD11, which causes decreased tumor growth and invasiveness in DLBCL cell lines 137. In miR-144/451 deficient mice, Ding et al. found that the aged mice are susceptible to developing B-lymphoma 138. Mechanistically, knockout of miR-144/451 directly upregulates the expression of c-Myc gene in hematopoietic cells. Furthermore, the oncoprotein c-Myc inversely modulates miR-144/451 expression by directly binding to the miR-144/451 promoter region, creating a miRNA-Myc positive feedback loop to maintain high expression of c-Myc in B-lymphocytes 138. In addition, other studies have identified many other tumor-suppressive miRNAs like miR27b 139 and miR-145-3p 140, whose abnormal expression contributes to B cell related lymphomagenesis. In contrast, deletion of miR-15a and miR-16-1 in B cells results in clonal lymphoproliferative disorders by regulating the expression of genes controlling cell-cycle progression 141, indicating its oncogenic function in B cell related lymphomagenesis. Moreover, miR-217 overexpression in B cells promotes lymphomas, most likely by downregulating the expression of DNA repair genes and by stabilizing BCL-6 expression, which could enhance the susceptibility of these cells to oncogenic events 142. Consistently, increased levels of miR-217 have been found in BL and DLBCL cell lines. Medina et al. demonstrated that overexpression of miR-21 causes a pre-B malignant lymphoid-like phenotype 143. Once miR-21 is inactivated, the tumors is regressed completely in a few days. Further study showed that miR-21 suppresses expression of tumor suppressors like PTEN and PDCD4 as well as upregulation of the PI3K-AKT-mTOR pathway in human DLBCL cell lines 144.

Recently, Song et al. reported that ALKBH5, a m^6^A modulator, promotes DLBCL cells proliferation by upregulating translational regulatory lncRNA1. Therefore, knockdown ALKBH5 represses the cell viability of DLBCL cells 145. In MYC-deregulated B-cell lymphomas, inhibition of ALKBH5 also suppresses the growth of tumor cells by enhancing expression of MYC-repressed genes like SPI1 and PHF12^146^. Similar to the positive roles of m6A erasers in DLBCL, the increased expression level of METTL3 is found in DLBCL tissues and cell lines, and silencing METTL3 expression in DLBCL cells inhibits cell proliferation rate by abating the m^6^A methylation and total mRNA level of pigment epithelium-derived factor (PEDF) 147, indicating that inhibition of m^6^A methylation likely suppresses tumorigenic properties of DLBCL cells. The different studies show that both m^6^A erasers and writers promote tumor progression in DLBCL, although they function in an opposing way when regulating the m^6^A process. Altogether, these findings stress the complex roles of m^6^A modulators in DLBCL and suggest that ALKBH5 and METTL3 could be potential targets for B cell related malignancies.

### Epigenetics of NKs in tumor progression

NKs function as the first line of immunological defense against tumor initiation and progression. Their activities are largely dependent on the activation or inhibition of their receptors, like the NKG2D receptor. To evade NKs-mediated cytotoxicity, cancer cells develop multiple strategies to regulate NKs receptors. One study showed that gliomas with IDH1 and IDH2 mutation downregulate expression of NKG2D ligands (ULBP1 and ULBP3) by affecting DNA methylation, thus escaping NKs immune surveillance 148. When these ligands are re-expressed via 5-Aza treatment, NKs can attack glioma cells. Interestingly, the DNA methylation frequency of NKG2D promoter in patients with hepatocellular carcinoma (HCC) is higher than that in chronic hepatitis B patients and healthy controls, indicating that NKG2D promoter methylation can be used as a biomarker for detecting hepatitis B virus-associated HCC 149. Codo et al. reported that miR-20a, miR-93 and miR-106b can regulate the expression of NKG2D ligands like ULBP3, thus enhancing the NKs-mediated lysis of glioma cells 150. Another study showed that EZH2 regulates NKs-mediated cancer cell eradication via transcriptional repression of NKG2D ligands including ULBP1 and ULBP2 in HCC cells 151. Therefore, the inhibition of EZH2 leads to HCC cell eradication via NKs by upregulating expression of ULBP3 and ULBP4 in tumor cells. Furthermore, valproic acid, a histone deacetylase inhibitor, has been reported to inhibit NKs lytic activity against leukemic cells due to the decreased expression of NKG2D by inducing histone K9 hypermethylation and DNA methylation in NKG2D promoter 152. In addition, Hicks et al. reported that entinostat induces a significant increase in protein expression NKG2D, NKp30 and DNAM-1 in NKs, thus augmenting NKs-mediated tumor cell killing in multiple carcinoma types 153. Besides these activated receptors, KIRs, a kind of inhibitory receptor, are also influenced by epigenetic mechanisms, causing different responses of NKs to tumor cells. For instance, 5-Aza significantly increases the expression levels of KIRs and represses their cytolytic activity against human leukemic cells 71. However, Kübler et al. reported that low-dose and long-term treatment of 5-Aza enhances the anti-tumor response not by inducing common KIR expression but by promoting the differentiation of various NKs precursor subsets in humanized NSG mice of acute myeloid leukemia 154. This disparity indicates that the different effects of 5-Aza on KIR expression could arise from the dose used in different studies, with high doses of the demethylating agents showing cytotoxicity and lower doses mediating DNA hypomethylation.

The function of NKs is determined by secreting various cytokines like IFN-γ and toxic agents like perforin and GzmB. Therefore, the epigenetic modulations of cytokines or toxic agents also influence anti-tumor activity of NKs. In a mouse model of epithelial ovarian cancer, 5-Aza increases the percentage of active NKs in the TME, together with upregulation of type I IFN signaling, thus reducing tumor burden and extending survival 155. Similarly, MM-102 and OG-L002 inhibitors increase the expression levels of IFN-γ and TNF-α by targeting H3K4 and H3K27 in NKs 156. Furthermore, miR-27a* has been reported to silence perforin and GzmB expression by specifically binding to their 3' untranslated regions in both resting and activated NKs 75. Accordingly, knockdown of miR-27a* in NKs dramatically increases cytotoxicity in vitro and suppresses tumor growth in a human tumor xenograft model. miR-182 augments NKs cytotoxicity by promoting perforin-1 expression and increases cytotoxic activity of NKs-against HCC cells 157.

The downregulated expression of METTL3 is also observed in tumor-infiltrating NKs of HCC patients, supporting the critical role of METTL3 regulating the function of NKs in carcinogenesis. Indeed, depletion of METTL3 in NKs inhibits NK cell infiltration and function in the TME, causing accelerated tumor development and shortened survival in mice by lowering expression of SH2 domain-containing protein tyrosine phosphatase-2 78. In contrast, deletion of FTO in NKs strengthens the function of NKs by decreasing the mRNA stability of SOCS family genes, thus enhancing tumoricidal activity of NKs in melanoma and leukemia models 158. Ma et al. also reported that YTHDF2 is required for NKs function by forming a STAT5-YTHDF2 positive feedback loop. Inhibition of YTHDF2 in NK cells, therefore, impairs NKs antitumor activity in vivo 159. All in all, these studies support the idea that epigenetic modulations of activity and function of NKs significantly impact tumor progression.

### Epigenetics of macrophages in tumor progression

Macrophages are key innate immune cells in the TME that regulate multiple tumorigenic properties including primary tumor growth, vascularization and metastatic dissemination. Therefore, the abnormal epigenetics in regulating the polarization and function of macrophages interfere considerably with tumor progression. In murine and human melanoma specimens, TET2 expression is increased in tumor associated macrophages (TAM), indicating the oncogenic function of TAM via TET2 modification 160. Not surprisingly, abortion of TET2 in myeloid cells suppressed melanoma growth in vivo by shifting the immunosuppressive gene expression pattern in TAM to a proinflammatory one, thereby causing the downregulation of the immunosuppressive function 160. Intriguingly, when mouse BMDMs are co-cultured with mouse pancreatic adenocarcinoma tumor cells, several M1 macrophage related metabolic genes are hypermethylated, leading to a suppressed glucose metabolic status in M1 but not in M2 macrophages, which in turn promote migration and metastasis of tumor cells 161. Furthermore, this cell-cell interaction could be prevented by the pre-treatment of a DNMT inhibitor, implying that tumor-educated macrophages promote tumor metastasis in a DNA methylation-dependent manner. In the same mouse model, DNA hypomethylating drug decitabine induces an increase in a subset of tumor-infiltrating M2 macrophages, slowing down tumor growth 162. Its combinatory use with anti-PD1 antibody additionally inhibits tumor growth and prolongs mouse survival. In an immunosuppressive mouse model of aggressive high-grade serous ovarian cancer, Travers et al. found that the combination of 5-Aza and DFMO (an ornithine decarboxylase inhibitor) significantly decreases immunosuppressive cells such as M2 macrophages and increases tumor-killing M1 macrophages, resulting in decreased tumor burden and prolonged survival 163. When macrophages are depleted with a CSF1R-blocking antibody, the anti-tumor efficacy of 5-Aza and DFMO treatment is impaired due to decreased M1 macrophages in the TME.

Many microRNAs have been indicated in regulating the polarization and function of macrophages in tumor progression. For instance, cancer cell-derived exosomal miR-138-5p inhibits M1 macrophages polarization and promotes M2 macrophages polarization by repressing KDM6B expression in macrophages 164. Not surprisingly, Macrophages treated with exosomal miR-138-5p promote lung metastasis. In addition, snail-expressed tumor cell-derived miR-21 has been reported to suppress the expression of M1 macrophages markers and increase M2 macrophage specific markers, and knockdown of miR-21 in snail-expressing tumor cells attenuates snail-induced M2 macrophages polarization, angiogenesis and tumor growth 165. Yang et al. reported that miR-19a-3p inhibits the M2 macrophages polarization in the TME of 4T1 breast mouse model by downregulating expression of the FRA1 gene. The overexpression of miR-19a-3p, therefore, represses metastasis of 4T1 breast cancer cells by suppressing M2 macrophage function 166. Besides these microRNAs, RNA-associated regulators subtly control function of macrophages during tumor progression. Yin et al., reported that METTL3 is critical for macrophages in anti-tumor response. The deletion of METTL3 in macrophages impairs the YTHDF1-mediated translation of SPRED2, which enhances the activation of NF-kB and STAT3 through the ERK pathway, leading to increased tumor growth and metastasis 167. Furthermore, METTL3 deficiency suppresses TLR signaling-mediated macrophage activation by reducing degradation of IRAKM, which increases susceptibility to bacterial infection and enhancing tumor growth in mice 84. In contrast, deletion of METTL14 in macrophages predominantly impairs function of CD8^+^ T cells by upregulating expression of Ebi3, a subunit of both of the heterodimeric cytokines IL-27 and IL-35, leading to tumor progression 86, which indicates the crosstalk between different immune cells could impact tumor progression.

Likewise, accumulating evidence suggests that epigenetic modifications related to chromatin have a role in regulating the polarization and function of macrophages in tumor progression. For instance, USP24 increases the level of histone H3 acetylation in the promoters of IL-6 and NFKB1 by stabilizing HAT p300, which upregulates the expression of these genes in M2 macrophages, promoting the progression of lung cancer 168. The shortage of histone phosphorylation at the IL-12 promoter region and the enrichment of ERK1/2-dependent histone phosphorylation at the IL-10 promoter region causes the polarization of TAM toward a more immunosuppressive form, which might contribute to tumor growth 169. Joshi et al. showed that JQ1, a BET bromodomain inhibitor, disrupts the occupancy of bromodomain-containing protein 4 (BRD4) on promoters of arginase and other IL-4-dependent macrophage immunosuppressive genes in the TME. Therefore, the combination of JQ1 with a PI3K inhibitor delays tumor growth in syngeneic and spontaneous mouse cancer models 170. Interestingly, HDACs show an ambivalent effect on the regulation of gene expression in TAM, which differently influences tumor progression. SAHA, an HDAC inhibitor, regulates M2 macrophage polarization and function through alteration of histone acetylation, thereby promoting tumorigenic properties in prostate cancer cells 171. In contrast, in estrogen receptor-negative mammary tumors in MMTV-polyoma middle T (PyMT) mouse model, the use of SAHA together with triterpenoid significantly delays the initial development of tumors partially due to decreased macrophage infiltration into the mammary gland 172, indicating that HDAC inhibition contributes to tumor repression in PyMT mouse model. Similarly, in a macrophage-dependent autochthonous mouse model of breast cancer, Guerriero et al. demonstrated that TMP195, a selective class IIa HDAC inhibitor, reduces tumor burden and pulmonary metastases by mediating macrophage phenotypes 173. Furthermore, TMP195 enhances the recruitment and differentiation of immunostimulatory macrophages in the TME. Therefore, the use of TMP195 with chemotherapy or immune checkpoint blockade strikingly induces the tumor reduction, supporting the promoting effects of HDACs on tumor progression. Altogether, these findings suggest that epigenetic regulators are potential targets for treating tumor by influencing the polarization and function of macrophages in tumor progression. Nevertheless, more experimental evidence is needed to unravel the mechanism by which inhibition of some epigenetic regulators causes a more aggressive phenotype in certain cancer types.

### Epigenetics of neutrophils in tumor progression

Neutrophils are multifaceted innate immune cells that are present in many different types of cancers like renal cell carcinoma (RCC), CRC and melanoma. The crosstalk between neutrophils and cancer cells is complicated. Growing evidence shows the important role of epigenetic mechanisms for the recruitment of neutrophils in the TME. For example, the neutrophil-enriched pancreatic cancer clones increase CXCL1 expression that is mediated at the epigenetic level via a combination of more accessibility of the promoter region, enrichment of the active H3K4me3 histone marker and activity of c-Myc that governs CXCL1 expression 174. The increased CXCL1 expression in turn recruits neutrophil cell infiltration into pancreatic ductal adenocarcinoma (PDA), generating an immunosuppressive microenvironment in PDA 175. In a preclinical model of clear cell renal cell carcinoma, massive expression of inflammation-related genes including CXCL1 is transcriptionally activated by epigenetic mechanisms such as DNA demethylation and super-enhancer formation 176. The amplification of cancer-cell-intrinsic inflammation during tumor progression promotes neutrophil-dependent lung metastasis. Therefore, the blockage of binding of BRD4 and SEs at genomic loci via a BET inhibitor represses neutrophil-dependent lung metastasis by aborting CXCL gene expression 176.

Since CXCL8 regulates the chemotaxis of human neutrophils, CXCL8 produced by cancer cells has been reported to promote aggressive phenotypes in multiple cancer types, including melanoma and lung cancer, by strengthening the recruitment of neutrophils in the TME 177. Manfroi et al. further showed that the inhibition of DNA methylation using decitabine and the blockage of histone deacetylase via TSA promotes the production of CXCL8 in CXCL8^+^ DLBCL cells but not in CXCL8^-^ DLBCL cells 178. This disparity of CXCL8 expression partially explains why CXCL8^+^ DLBCL cells are more aggressive than CXCL8^-^ DLBCL cells 179. In nasopharyngeal carcinoma cells the phosphorylated MSL1 via PKB augments the transcription of CD276 by increasing histone H4 Lys16 acetylation at the promoter region of CD276^180^. Therefore, upregulated CD276 contributes to the recruitment of neutrophils into TME, thereby promoting tumorigenesis. In addition, IL-8 plays a pivotal role in chemotaxis of neutrophils. Deletion of METTL3 in papillary thyroid carcinoma promotes recruitment of tumor-associated neutrophils by eliciting secretion of IL-8, therefore supporting tumor progression 181. Interestingly, neutrophils can produce exosomes with iRNA-17560, which enhances the expression of FTO in breast cancer cells. The increased FTO strengthens the stability of ZEB1 mRNA transcripts, which results in chemoresistance and epithelial-mesenchymal transition of tumor cells, indicating neutrophils control tumor progression by impacting the RNAs regulators in tumor cells. Altogether, these studies suggest that the crosstalk between neutrophils and cancer cells via different epigenetic mechanisms contributes to tumor progression.

Recently, one study reported that splenic neutrophils from β-glucan-trained mice can diminish tumor growth. Mechanistically, β-glucan enhances chromatin accessibility for these genes of the ROS-producing factors NCF1 and NCF2 as well as type I IFN signaling-related genes like IRF1and IFNAR1 in these neutrophils, resulting in neutrophils reprogramming toward an anti-tumor phenotype 182. Interestingly, β-glucan has been reported to regulate other innate immune cell differentiation via different epigenetic regulators like miR-9-5p 183. However, the way in which β-glucan influences the epigenome of neutrophils needs to be further investigated.

Based on the analysis of DNA methylation in neutrophils and lymphocytes among patients with breast cancer, high levels of DNA methylation-derived neutrophil-to-lymphocyte ratio are associated with a poor outcome 184, indicating that these patients can benefit from inhibition of DNA methylation. Another study also reported that knockdown of UHRF1 reduces the methylation level of RIP3 promoter and induces the expression of RIP3, which contributes to tumor suppression by enhancing the infiltration of neutrophils into the tumor site 185. Interestingly, apoptotic cancer cells release epigenetically regulated cytokines including CXCL10 and CCL2, enhancing nucleic acid-elicited phagocytosis of dying cancer cells by neutrophils, providing alternative approaches for neutrophil-based anticancer therapy 186. Collectively, the epigenetic regulation of neutrophils or cancer cells also generates a favorable immunological TME, thereby contributing to tumor repression.

### Epigenetics of DCs in tumor progression

During tumor progression, epigenetic alterations that change DCs maturation and function are thought to impair an effective anti-tumor immune response. For instance, miR-22 suppresses the maturation and antigen presentation function of DCs by directly targeting the 3'UTR of p38, an important regulator in controlling DCs activity 187. Further analysis showed that miR-22 overexpression in DCs blocks their tumor-suppressing ability, while inhibition of miR-22 could converse this phenotype and promote the curative effect of DCs-based immunotherapy 187. Similarly, miR-155 has been shown to modulate DCs function by diminishing PRC2 recruitment and H3K27me3 presence at C-C chemokine receptor type 7 gene locus 188. The miR-155 deficiency in DCs alleviates their maturation, migration ability, and cytokine production, and thus diminishes the effectiveness of DCs-based immunotherapy for breast cancer in mouse model 188. Indeed, the DCs vaccine with miR-155 overexpression results in enhanced anti-tumor immunity against established breast cancers in mice, by promoting DCs maturation and migration 189. Interestingly, this study also showed that miR-155 expression can be inhibited by abundant IL-6 and IL-10 in the TME. The overproduction of these two cytokines has been indicated in many other cancer types, indicating that increasing miR-155 expression by interfering with IL-6 and IL-10 could be an alternative approach for other type of cancer. In addition, other RNA epigenetic modulators have been reported to regulate tumor progression by targeting DCs function. Han et al. demonstrated that YTHDF1 specifically binds to these transcripts encoding lysosomal cathepsins in DCs, and influences antigen degradation in DCs lysosomes 107. Loss of YTHDF1 enhances the cross-presentation of tumor antigens by decreasing translational efficiency of cathepsins, thus creating a favorable antitumor immune microenvironment. Not surprisingly, therapeutic efficacy of PD-L1 is enhanced in YTHDF1 knockout mice.

In addition, SATB1 governs the differentiation of inflammatory DCs by epigenetically regulating MHC II expression by driving RBPJ occupancy of the H2-Ab1 promoter, thereby affecting tumor progression 190. Shi et al. showed a strong interaction between DNMTs and immune genes associated with the infiltration of DCs in CRC 191, indicating the important role of DNMTs in DCs infiltration during tumor progression. These studies suggest that the abnormal epigenome influences tumor progression by interfering with DCs function or differential, and thus epigenetic regulators could be potential targets for cancer therapy.

Indeed, emerging evidence shows that epigenetic-related compounds dampen tumorigenic properties via DCs. For instance, FOXM1, a regulator of DC maturation, is epigenetically regulated by H3K79me2 in the TME. Increased H3K79me2 enrichment is observed at the FOXM1 promoter in both bone marrow-derived DCs from tumor-bearing mice and TME 192. The inhibition of the H3K79 methyltransferase DOT1L via EPZ004777 not only decreases enrichment of H3K79me2, but also attenuates the expression of FOXM1, which partially decelerates tumor growth by enhancing function of bone marrow-derived DCs 192. HDAC inhibitors have been reported to enhance the percentage of immunogenic DCs, generating a favorable immunological TME. For instance, HDAC inhibitors can suppress the conversion of immunogenic DCs to immunosuppressive DCs, suggesting a role for HDACs in the generation of an immunosuppressive TME 193. Similarly, low-dose combination of two FDA-approved epidrugs, 5-Aza (A) and romidepsin (R), with IFNα2 (ARI) suppresses the aggressiveness of CRC by augmenting DCs function 194. Further analysis indicated that ARI-induced histone methylation and acetylation alterations epigenetically influence the promoters of IFN-stimulated genes in DCs, endowing DCs with a marked migratory capability. Furthermore, in a mouse model of breast cancer, treatment with epigenetic modulators (DZNeP and 5-Aza-dC) alone had limited effects on the production of CXCL9 and CXCL10 by DCs. But treatment with the modulators combined with anti-PD1 antibody strikingly increases CXCL9 and CXCL10 expression in DCs, resulting in a significant delayed tumor growth and prolonged mouse survival 195. Similarly, IFN-α combined with epigenetic modulators (azacitidine and romidepsin) strongly inhibits invasive signaling pathways in both metastatic cells and cancer stem cells of CRC, which improves DCs phagocytosis of cancer cells by triggering cell death with immunogenic features 196.

## Conclusions and perspectives

Since epigenetic modifications can alter gene expression in a heritable-dependent manner, they play an essential role in maintaining cellular homeostasis by controlling the expression of genes. Interestingly, different epigenetic modifications could have a similar function in regulating gene expression. Both DNA methylation and histone deacetylation can repress gene transcription. Conversely, both DNA demethylation and histone acetylation activate gene transcription. Emerging evidence indicates that histone acetylation could induce DNA hypomethylation which enhances the chance of trans-differentiation in adipocytes, indicating crosstalk between histone modifications and DNA methylation in regulating gene expression. Recently, an increasing number of new epigenetic modifications have been identified. For instance, lactate, an energy source and metabolic by-product, serves in the epigenetic modification of histone lysine residues, which directly stimulates gene transcription from chromatin. Under hypoxia and bacterial challenges, histone acetylation directly regulates gene expression to promote M2 characteristics in the late phase of M1 macrophage polarization to ultimately achieve a homeostatic response 197. In addition, lysine isobutyrylation has been identified as a new histone modification mark 198. However, further studies are needed to establish whether these modifications with similar functions in regulating gene expression can work together or independently regulate gene expression in a temporal and spatial-dependent manner.

The differentiation, development and function of immune cells is tightly maintained by different genes. The abnormal expression of these genes often impairs immune homeostasis, thus resulting in human diseases ranging from cardiovascular diseases to cancer. Since epigenetic modifications play critical roles in regulating gene expression, epigenetic dysfunction has been connected to various human pathologies by altering related genes expression. For instance, RELA is an important TFs for regulating immune response, and the abnormal epigenetic modulations of RELA are considered as the potential reason for atherosclerosis 199. More broadly, the aberrant epigenetic modifications in various immune cells have been widely considered as important mechanisms for tumor initiation, progression and metastasis. Several epigenetic drugs like panobinostat (an HDAC inhibitor) and tazemetostat (an EZH2 inhibitor) have been approved to treat lymphoma and epithelioid sarcoma in the clinic. In particular, immune cells are the premise for cancer immunotherapy, so the epigenetic dysfunction among various immune cells closely influences the efficacy of cancer immunotherapy. For instance, DNMT3a-mediated de novo DNA methylation in activated CD8^+^ T cells is considered a mechanism that restricts the efficacy of cancer immune checkpoint blockade therapy, highlighting the ability of DNA-demethylating agents to enhance immune checkpoint blockade-mediated T cell response 200. Collectively, epigenetic regulators provide attractive targets for treating human diseases by regulating the expression of immune related genes.

It should be noted that epigenetic modulations of genes lack specificity in most cases, therefore targeting them could interfere with the expression of various genes. The epigenetic drugs targeting HDACs or DNMTs often alter global acetylation and methylation, respectively, which inevitably influences on the expression of numerous related genes. The unintended consequences increase cytotoxicity of these drugs, thus limiting their therapeutical efficacy. Furthermore, the epigenetic modulations of genes are cell type- dependent, which further increases the unintended consequences of these epigenetics targets in vivo. More importantly, epigenetic drugs could also regulate the epigenetic landscape of normal cells and affect their cellular homeostasis. The identification of gene-specific or cell type-specific epigenetic deregulation could reduce these off-target effects. Accumulating evidence suggests that epigenetic alterations could be the reason for acquired resistance to other cancer therapies; aberrant epigenetic modulations have been observed in some cancer cells, indicating primary resistance to epigenetic drugs. Furthermore, acquired resistance is also observed in the epigenetic drug-treated cancer cells. Therefore, identification of potential biomarkers of response could provide better guidelines for epigenetic therapies. The solutions to these unsolved issues will contribute to clinical applications of epigenetic drugs not only for cancer treatment but also for other human diseases.

## Figures and Tables

**Table 1:** The type of histone modifications

MOD	Modification and site	Abbreviation	Role
Ac	Histone 2A lysine 5 acetylation	H2AK5ac	Transcriptional activation
	Histone 2B lysine 5 acetylation	H2BK5ac	Transcriptional activation
	Histone 2B lysine 12 acetylation	H2BK12ac	Transcriptional activation
	Histone 2B lysine 15 acetylation	H2BK15ac	Transcriptional activation
	Histone 2B lysine 20 acetylation	H2BK20ac	Transcriptional activation
	Histone 3 lysine 4 acetylation	H3K4ac	Transcriptional activation
	Histone 3 lysine 14 acetylation	H3K14ac	Transcriptional activation
	Histone 3 lysine 18 acetylation	H3K18ac	Transcriptional activation
	Histone 3 lysine 23 acetylation	H3K23ac	Transcriptional activation
	Histone 3 lysine 36 acetylation	H3K36ac	Transcriptional activation
	Histone 3 lysine 9 acetylation	H3K9ac	Transcriptional activation
	Histone 3 lysine 27 acetylation	H3K27ac	Transcriptional activation
	Histone 3 lysine 56 acetylation	H3K56ac	Histone deposition
	Histone 4 lysine 5 acetylation	H4K5ac	Transcriptional activation
	Histone 4 lysine 8 acetylation	H4K8ac	Transcriptional activation
	Histone 4 lysine 16 acetylation	H4K16ac	Transcriptional activation
	Histone 4 lysine 12 acetylation	H4K12ac	Histone deposition
	Histone 4 lysine 91 acetylation	H4K91ac	Histone deposition
Me	Histone 3 lysine 4 methylation	H3K4me	Transcriptional activation
	Histone 3 lysine 9 methylation	H3K9me	Transcriptional repression
	Histone 3 lysine 27 methylation	H3K27me	Transcriptional repression
	Histone 3 lysine 36 methylation	H3K36me	Transcriptional activation
	Histone 3 lysine 79 methylation	H3K79me	Transcriptional activation
	Histone 3 arginine 2 methylation	H3R2me	Transcriptional activation
	Histone 3 arginine 8 methylation	H3R8me	Transcriptional activation
	Histone 3 arginine 17 methylation	H3R17me	Transcriptional activation
	Histone 3 arginine 26 methylation	H3R26me	Transcriptional activation
	Histone 4 arginine 3 methylation	H4R3me	Transcriptional activation
	Histone 4 lysine 20 methylation	H4K20me	Transcriptional repression
P	Histone 2A serine 1 phosphorylation	H2AS1ph	Mitosis
	Histone 2A threonine 120 phosphorylation	H2AT120ph	Mitosis, transcriptional activation
	Histone 2A.X serine 139 phosphorylation	H2A.XS139ph	DNA repair
	Histone 2B serine 14 phosphorylation	H2BS14ph	Apoptosis
	Histone 3 threonine 6 phosphorylation	H3T6ph	Transcriptional activation
	Histone 3 threonine 3 phosphorylation	H3T3ph	Mitosis, DNA repair
	Histone 3 serine 10 phosphorylation	H3S10ph	Mitosis, DNA repair
	Histone 3 threonine 11 phosphorylation	H3T11ph	Mitosis, DNA repair
	Histone 3 serine 28 phosphorylation	H3S28ph	Mitosis, DNA repair
	Histone 3 threonine 45 phosphorylation	H3T45ph	DNA replication
	Histone 4 serine 1 phosphorylation	H4S1ph	Mitosis, transcriptional activation
Ub	Histone 2A lysine 119 ubiquitination	H2AK119ub	Transcriptional repression
	Histone 2B lysine 120 ubiquitination	H2BK120ub	Transcriptional activation
	Histone 3 lysine 23 ubiquitination	H3K23ub	Maintenance of DNA methylation
Ser	Histone 3 glutamine 5 serotonylation	H3Q5ser	Transcriptional activation
La	Histone 3 lysine 18 lactylation	H3K18la	Transcriptional activation
	Histone 4 lysine 12 lactylation	H3K18la	Transcriptional activation
Cr	Histone 3 lysine 9 crotonylation	H3K9cr	DNA repair
	Histone 3 lysine 18 crotonylation	H3K18cr	Transcriptional activation
	Histone 3 lysine 27 crotonylation	H3K27cr	Transcriptional activation
	Histone 3 lysine 4 crotonylation	H3K4cr	Transcriptional activation
	Histone 3 lysine 14 crotonylation	H3K14cr	Transcriptional activation

MOD: modification, Ac: acetylation, Me: methylation, P: phosphorylation, Ub: ubiquitination, Ser: serotonylation, La: lactylation, Cr crotonylation

## Abbreviations

TSGs: tumor suppressor genes
Th1: T helper 1
TME: tumor microenvironment
5hmC: 5-hydroxymethylcytosine
5fC: 5-formylcytosine
5caC: 5-carboxylcytosine
DNMT1/3α/3β: DNA methyltransferase 1//3α3β
HATs: histone acetyltransferases
HDACs: histone deacetylases
TFs: transcription factors
NAD: nicotinamide adenine dinucleotide
KMTs: lysine methyltransferases
PaRMTs: protein arginine methyltransferases
COMPASS: complex of proteins associated with SET1
ADMA: asymmetric dimethyl arginine
SDMA: symmetric dimethyl arginine
Ub: ubiquitin
INO80: inositol 80
NuRD: Mi-2/nucleosome remodeling and histone deacetylation
ISWI: imitation-switch
SWI/SNF: switching defective/sucrose non-fermenting
METTL3/4/16: methyltransferase 3/4/16
WTAP: Wilms' tumor 1-associating protein
VIRMA: Vir-like m^6^A methyltransferase associated
FTO: fat mass and obesity associated
ALKBH5: alpha-ketoglutarate-dependent dioxygenase AlkB homolog 5
YTHDC1/2: YTH N6-Methyladenosine RNA Binding Protein C1/2
YTHDF1/2/3: YTH N6-Methyladenosine RNA Binding Protein F1/2/3
IGF2BPs: insulin-like growth factor 2 mRNA-binding proteins
ncRNAs: non-coding RNAs
miRNAs: microRNAs
siRNAs: small interfering RNAs
piRNAs: PIWI-interacting RNAs
lncRNAs: long non-coding RNAs
NKs: natural killer cells
DCs: dendritic cells
Tregs: regulatory T cells
Cxxc1: Cxxc finger protein 1
PEDF: pigment epithelium-derived factor
SOCS: suppressors of cytokine signaling
CNS2: conserved noncoding sequence 2
GzmB: granzyme B
GC: germinal center
KIRs: killer Ig-like receptors
NCRs: natural cytotoxic receptors
M1: M1-like macrophages
M2: M2-like macrophages
LPS: lipopolysaccharides
MHC: major histocompatibility complex
CRC: colorectal cancer
DLBCL: diffuse large B-cell lymphoma cells
TAM: tumor associated macrophages
BRD4: bromodomain-containing protein 4
PyMT: polyoma middle T
RCC: renal cell carcinoma
CCR7: C-C chemokine receptor type 7
